# Total and respirable dust exposures among carpenters and demolition workers during indoor work in Denmark

**DOI:** 10.1186/s12995-016-0134-5

**Published:** 2016-09-20

**Authors:** Lilli Kirkeskov, Dorte Jessing Agerby Hanskov, Charlotte Brauer

**Affiliations:** 1 Department of Occupational and Environmental Medicine, Bispebjerg Hospital, Copenhagen, Denmark; 2 Department of Occupational and Environmental Medicine, Unit of Social Medicine, Frederiksberg Hospital, Nordre Fasanvej 57, 2000 Copenhagen F, Denmark

**Keywords:** Total dust, Respirable dust, Respirable crystalline silica dust, Construction workers, Exposure assessment

## Abstract

**Background:**

Within the construction industry the risk of lung disorders depends on the specific professions probably due to variations in the levels of dust exposure, and with dust levels depending on the work task and job function. We do not know the extent of exposure in the different professions or the variation between the different work tasks. The purpose of this study was therefore to assess if there were differences in dust exposure between carpenters and demolition workers who were expected to have low and high dust exposure, respectively.

**Methods:**

Through interviews of key persons in the construction industry the most common work tasks were selected, and the concentration of dust during these tasks (indoors) were measured by personal sampling varying between 4 and 6 h of a working day. In total 38 measurements of total dust, and 25 of respirable dust on seven different work tasks were carried out for carpenters and 20 measurements of total dust, 11 of respirable dust and 11 of respirable crystalline silica dust on four different works tasks for demolition workers. Dust measurements were tested for differences using linear regression, t-test and one-way ANOVA.

**Results:**

For carpenters the geometric mean for all the measurements of total dust was 1.26 mg/m^3^ (geometric standard deviation 2.90) and the respirable dust was 0.27 mg/m^3^ (geometric standard deviation 2.13). For demolition workers the geometric mean of total dust for all the measurements was 22.3 mg/m^3^ (geometric standard deviation 11.6) and the respirable dust was 1.06 mg/m^3^ (geometric standard deviation 5.64).

The mean difference between total dust for demolition workers and carpenters was 11.4 (95 % confidence interval 3.46–37.1) mg/m^3^. The mean difference between respirable dust for demolition workers and carpenters was 3.90 (95 % confidence interval 1.13–13.5) mg/m^3^.

Dust exposure varied depending on work task for both professions. The dustiest work occurred during demolition, especially when it was done manually.

Only few workers used personal respiratory protection and only while performing the dustiest work.

**Conclusions:**

This study confirmed that the exposure to dust and especially total dust was much higher for demolition workers compared to carpenters.

**Trial registration:**

(ISRCTN registry): The study is not a clinical trial and are thus not registered.

## Background

Workers in the construction industry represent 6 % of employees in Denmark and are still daily or regularly exposed to dust. Different kinds of dust exposure including crystalline silica dust exposure occur commonly in the construction industry and are known causes for developing lung disorders.

Dust exposure in the workplace can be associated with chronic obstructive pulmonary disease (COPD) and an increased risk of COPD for workers exposed to dust has been found [1].

Earlier studies have shown differences in the risk to be hospitalised due to diseases in the lower respiratory system among different professions within the construction industry in Denmark [2, 3] which may be caused by differences in dust exposures. A cross-sectional study of demolition workers found an increased prevalence of self-reported lung symptoms (cough and sputum daily for three month per year for two years) for demolition workers (Odds ratio, OR 2.0, 95 % confidence interval, CI 1.0–3.9) and carpenters (OR 1.7, 95 % CI 0.9–3.3) compared to a group of non-exposed hospital porters and an increased OR 2.7, 95 % CI 1.3–2.7 for forced expiratory volume in one second below lower limit of normal ((FEV_1_ < LLN) for demolition workers compared to carpenters [4].

The construction industry includes many professions and many different work tasks for each profession, and the transient nature of construction sites makes it more difficult to accurately characterize exposures among construction workers. The best way to determine an adverse effect on the respiratory system due to dust exposure for individual workers is to directly measure the dust exposure and relate it to the lung function parameter for the individual worker over long periods (years). However, it is expensive and time-consuming to measure prospectively, especially for diseases that take more than a decade to develop.

The purpose of this study was to assess whether there were differences in dust exposure between two types of construction workers: carpenters and demolition workers. Based on previous studies and practical knowledge carpenters and demolition workers were chosen because these professions in the construction industry would probably have low and high exposures to dust, respectively [5–7].

In Denmark, carpenters are skilled construction workers. Their primary work tasks include installation of gypsum, doors, windows, rafters and roofs, floorings made of wood, ceilings and insulation. Demolition workers are unskilled construction workers. Their job includes manual and mechanical demolition tasks on whole or large parts of buildings, including management of waste (building material) and cleaning after demolition.

## Methods

### Measurements of exposure

Interviews of key persons from the trade union in the construction sector were performed to identify the most common work tasks. The work tasks were categorized as installation of gypsum, ceilings, floorings, windows and doors, insulation, and ‘other work tasks’ for carpenters. For demolition workers the work tasks were categorized as manual demolition, mechanical demolition, waste management and ‘other work tasks’.

The measurements were only carried out on indoor work tasks because of the outdoor measurement-uncertainties due to changing wind conditions. The working environment council of the construction industry participated in finding construction companies performing the requested tasks in two areas of Denmark (Copenhagen and Aarhus). Among companies accepting to participate we selected companies of different sizes and different work places.

A total of 11 companies (and 11 workplaces) were selected for carpenters. Measurements of total dust (TD) were carried out on 38 carpenters (1 measurement per worker) and of respirable dust (RD) on 17 carpenters (25 measurements) (1–2 measurements per worker).

Measurements of TD, RD and respirable crystalline silica dust (RCS) were carried out in 5 companies (5 workplaces) for demolition workers. Measurements of TD were carried out on 16 demolition workers. For seven workers RD was measured at the same time.

The measurements were made after identification of the most relevant work tasks, included measurements for each work task. Furthermore the measurements were conducted over an entire working day (excluding pauses) or as long as the task was done. Workplaces were not selected by level of dust (worst cases) but chosen if the work task was carried out.

The measurements were spread throughout the year: 11 % (winter), 22 % (spring), 30 % (summer), and 34 % (autumn).

In connection with the dust measurements it was registered if the workers used personal respiratory protective equipment, the use of local exhaust or other dust-reducing measures.

Performed measurements corresponded to a total of 255 working hours for carpenters and 113 working hours for demolition workers (pauses excluded).

### Work place monitoring of airborne dust

The concentration of TD was measured by personal sampling during 4 to 6 h of a working day. Sampling was performed using 37 mm Millipore filter cassettes mounted with 0.8 μm Mixed Cellulose Ester Membrane filters (fa. Millipore AAWOP) mounted in closed face Millipore field monitors with a 5.6-mm inlet at 1.9 L/min (inlet velocity = 1.25 m/s) (SKS Inc. Pennsylvania, U.S). The field monitor was placed in the breathing zone just below the collarbone. The inlet pointed downward. Flow rates were adjusted and controlled in the field before and after sampling by Porter Flow Meters (Porter Air Flow meters, model F65, measurement range 0.5-3 L/min.) calibrated against certified primary references. Air velocity in inlet was adjusted to 1.25 m/s according to Danish legislation. Each series of sampling were controlled and corrected towards two blank field samples. Conditioning of filters were performed prior and after sampling under controlled climatic conditions (temperature 22–28 °C and humidity 40–52 % RH). We used sampling pumps SKC 224 and SKC SideKick.

RD was sampled with modified Higgins and Dewell cyclones [8]. The 50 % aerodynamic diameter cut-point for collection efficiency was 5 μm, and the volumetric air sampling rate 2 L/min according to the Danish Working Environment Authority Guidelines [9]. The respirable fraction was collected on 37-mm diameter 1-μm membrane filters.

The collected mass of dust on the filters was determined gravimetrically. The limit of detection, LOD was calculated as three times the standard deviation (SD) of the blanks (30 μg) and the relative standard deviation of the method was 10 %.

Respirable crystalline silica dust (RCS) was analysed according to National Institute of Occupational Safety and Health (NIOSH) method 7602 (modified, accredited) [10]. The limit of detection was 5 μg. Both sampling and gravimetric analysis were carried out by Eurofins Miljø A/S. (Eurofins Miljø are accredited by The Danish Accreditation Fund); DANAK and testing was performed in accordance with the national and international standards as approved by DANAK, (DANAK Reg. No. 168 (sampling) and Reg. No. 522 (gravimetry)).

#### Data analysis

Statistical analyses were conducted using SPSS software (IBM SPSS Statistics, version 22, IBM Corp. 2013). We used a logarithmic scale for the graphic presentation because the distribution was skewed with a long right trail, and normalized by log-transformation. For measurements below the lowest limit of detection and above maximum quantification the detection limit/the maximum quantification values were used in the calculations. These values account for 4.8 and 1.9 % of the measurements, respectively.

The exposure levels were described by arithmetic means (AM), geometric means (GM), and geometric standard deviations (GSD) for each occupation and for each task.

The average exposure over an eight hour time period (normal work shift), 8-h TWA was calculated as: 8-h TWA = ∑_*i* = 1_^*n*^*CiTi*/8 h, where Ci = concentration during the i^th^ interval, and Ti= duration of the i^th^ interval.

Histograms, QQ-plots, and tests of skewness showed lognormal distribution and the dust concentrations were therefore lognormal transformed before statistical analysis. Differences in TD and RD between the two occupations, between work task measurements within the two occupations and across occupations were tested using t-test, one-way ANOVA and linear regression. Log(TD) and log(RD) were used in the models. Correlations between TD and RD for the two professions were tested (Spearman’s correlation).

The relationship between dust concentration and occupation was also investigated using linear regression analysis with carpenters used as reference category and adjusted for seasonal variations. Season was in the analysis divided in winter/spring and summer/autumn with summer/autumn used as reference category. Analyses were made on log-transformed data.

## Results

### Carpenters

The GM of all the measurement of TD was 1.26 mg/m^3^ ranging from minimum 0.08 mg/m^3^ when installing material of iron to maximum 8.40 mg/m^3^ when stiffening of beams. Within the work task installation of gypsum the concentration of TD varied between 1.40 mg/m^3^ and 7.00 mg/m^3^ (Table 1). The main reasons for dust exposure among carpenters was especially use of hand-held high-speed tools, grinding, lack of local exhaust ventilation, lack of cleaning during a work task, lack of cleaning before the next occupation started their work and dust exposure from other occupations who worked at the same time. None of the carpenters used airway protection during the work.

**Table 1:** Measurements of dust for carpenters (GM, GSD, and AM)

	Number of measurements	Average sampling time (min)	Sampling time (min-max) (min)	Number of <LOD	GM mg/m^3^ GSD		AM mg/m^3^	min-max
Total dust								
Gypsum, installation	8	346	296–401	0	2.22	1.77	2.63	1.40–7.00
Ceilings, installation	12	281	211–292	0	0.86	3.92	1.56	0.08–4.00
Installation of windows, doors	5	308	262–433	0	0.61	1.78	0.69	0.32–1.00
Installation of floors	4	240	233–245	0	1.00	1.80	1.14	0.52–1.70
Insulation	5	232	100–289	0	1.59	1.13	1.60	1.30–1.80
Wood work	2	219	101–336	0	1.57	1.31	1.60	1.30–1.90
Other (stiffening of beams)	2	206	167–244	0	5.50	1.82	6.00	3.60–8.40
Total	38	280	100–401	0	1.26	2.90	1.94	0.08–8.40
Respirable dust								
Gypsum, installation	8	213	185–250	0	0.57	1.75	0.67	0.27–1.50
Ceilings, installation	0	-	-	0	-	-	-	-
Installation of windows, doors	5	205	196–239	1	0.18	1.75	0.20	<0.07–0.31
Installation of floors	2	244	239–249	0	0.24	1.22	0.25	0.20–0.30
Insulation	3	200	177–247	1	0.15	1.67	0.17	<0.09–0.30
Wood work, milling and cutting	5	198	187–248	1	0.16	1.42	0.17	<0.09–0.24
Other	2	202	196–208	0	0.42	1.85	0.46	0.27–0.65
Total	25	210	177–250	3	0.27	2.13	0.37	<0.09–1.50

*Notes*: *GM* geometric mean, *GSD* geometric standard deviation, *AM* arithmetic mean, *<LOD* below level of detection

The calculated 8-h-TWA for TD was 1.07 mg/m^3^ for all the measurements. None of the calculated 8-h-TWA for the individual measurements (0.04 to 4.27 mg/m^3^) exceeded the Occupational Exposure Limit (OEL) of 10 mg/m^3^ for TD.

The GM of RD was 0.27 mg/m^3^ ranging from no detectable RD during wood work to a maximum of 1.50 mg/m^3^ when installing gypsum on walls. The calculated 8-h-TWA for RD was 0.16 mg/m^3^ for all the measurements. None of the calculated 8-h-TWA for the individual measurements (0.01 to 0.33 mg/m^3^) exceeded the OEL of 5 mg/m^3^ for RD.

**Fig. 1:**
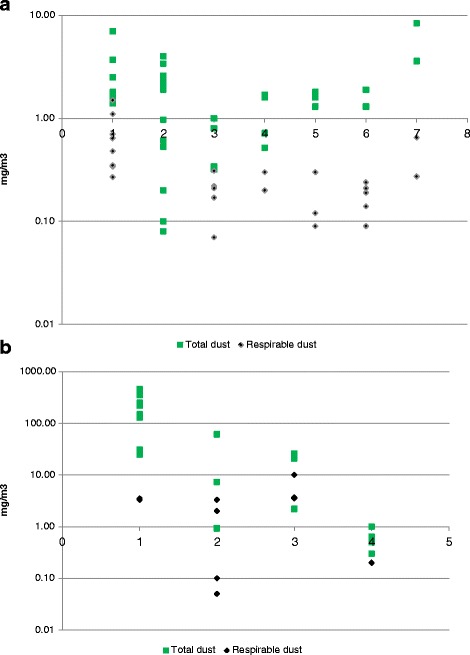
**a**. Task-specific measurements of total and respirable dust among carpenters (mg/m^3^). Note: 1: installation of gypsum; 2: installation of ceilings; 3: installation of windows and doors; 4: installation of floors; 5: insulation; 6: woodwork; 7: other work tasks. **b**. Task-specific measurements of total and respirable dust among demolition workers (mg/m^3^). Note: 1: manual demolition; 2: mechanical demolition; 3: waste management; 4: other work tasks

**Fig. 2:**
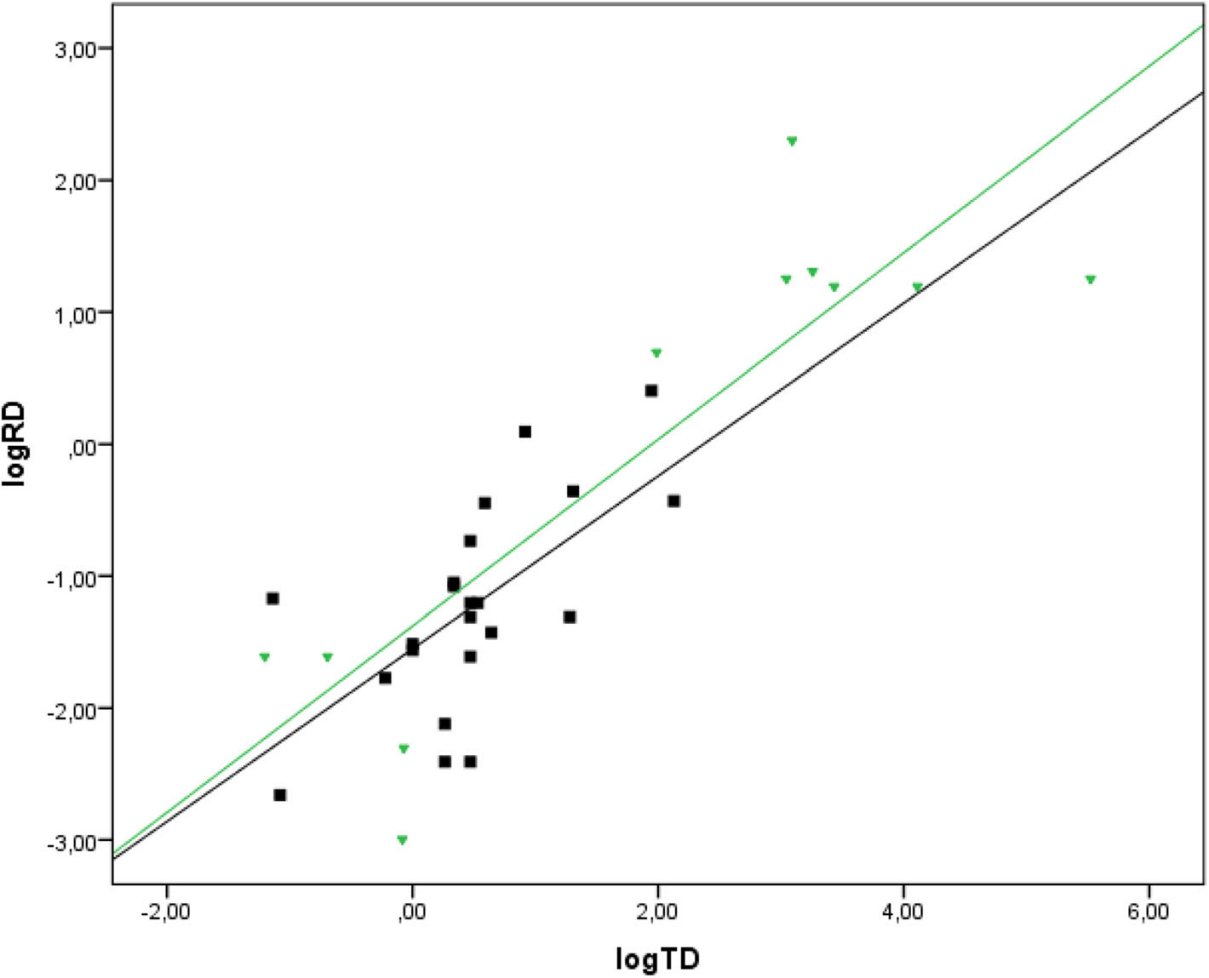
Correlation between total dust (log TD) and respirable dust (log RD) for carpenters (*N* = 21, black square), Spearman correlation, *r*_s_ = 0.64 and demolition workers (*N* = 11, green triangle), Spearman correlation, *r*_s_ = 0.69

Figure 1a shows the dust concentrations for TD and RD for carpenters divided in different work task. The dust concentrations differed comparing the work tasks for carpenters (F = 2.39, df = 37, *p* =0.05) for TD, and (F = 5.47, df = 24, *p* = 0.003) for RD (Fig. 2a). The difference between the work tasks for RD disappeared when the job tasks: ‘installing gypsum’ and ‘other work tasks’ (stiffening of beams) were excluded from the analysis (F = 0.48, df = 14, *p* = 0.70).

TD was moderate correlated with RD (Spearman’s correlation *r*_s_ = 0.64), Fig. 2.

### Demolition workers

The measurements showed GM of all the measurements of TD of 22.3 mg/m^3^ ranging between 0.30 mg/m^3^ for installation of scaffolding and support of ceilings and > 460 mg/m^3^ for manual demolition (Table 2). In general, mechanical demolition showed lower dust concentrations (4.4 mg/m^3^) compared to manual demolition (177 mg/m^3^). The concentrations were lowest during mechanical demolition, good ventilation, and/or use of water. During manual demolition using high-speed tools, dry-cutting, waste management and working indoors without sufficient ventilation the exposure was high. Waste management led to a high exposure to TD, RD and RCS. Demolition workers only used respiratory protection during manual demolition.

**Table 2:** Measurements of dust for demolition workers (GM, GSD, and AM)

	Number of measurements	Average sampling time (min)	Sampling time (min-max) (min)	Number of <LOD	GM mg/m^3^	GSD	AM mg/m^3^	min-max
Total dust								
Manual demolition	8	119	90–264	0	138	2.42	227	31.0–> 460
Mechanical demolition	4	207	117–287	0	4.42	7.41	17.5	0.92–61.0
Waste management	4	163	123–257	0	22.9	3.24	23.0	21.0–26.0
Other	4	181	108–255	0	0.39	1.44	0.40	0.30–0.50
Total	20	158	90–287	0	22.3	11.6	108	0.92–> 460
Respirable dust								
Manual demolition	2	118	117–118	0	3.40	1.03	3.40	3.30–3.50
Mechanical demolition	4	207	117–287	0	0.43	6.18	1.40	0.05–3.30
Waste management	3	131	123–257	0	5.06	1.62	5.73	3.50–10.0
Other	2	116	108–124	0	0.20	1.00	0.20	-
Total	11	143	108–287	0	1.06	5.64	2.71	0.05–10.0
Respirable silica dust								
Manual demolition	2	118	117–118	0	0.69	1.03	0.69	0.67–0.71
Mechanical demolition	4	207	117–287	0	0.09	6.04	0.23	0.02–0.45
Waste management	3	131	123–257	0	0.15	4.77	0.35	0.06–0.92
Cleaning	2	116	108–124	2	nd.	-	nd.	-
Total	11	143	108–287	2	0.12	5.25	0.31	nd.–0.92

*Notes*: *GM* geometric mean, *GSD* geometric standard deviation, *AM* arithmetic mean, *nd* not detectable, *<LOD* below level of detection

The calculated 8-h-TWA for TD was 17.8 mg/m^3^ for all the measurements. For 40 % of the measurements the calculated 8-h-TWA (0.06 to >86 mg/m^3^) exceeded the OEL of 10 mg/m^3^ for TD.

In total 11 measurements of RD and RCS were made. The measurements showed GM for RD concentrations of 1.06 mg/m^3^ (0.10–10 mg/m^3^), and for RCS of 0.12 mg/m^3^ [<0.01 (no detectable crystalline silica) to 0.92 mg/m^3^]. The lowest concentrations were shown for mechanical demolition and the highest for manual demolition.

The calculated 8-h-TWA for RD was 1.40 mg/m^3^ for all the measurements. Only one of the calculated 8-h-TWA for the individual measurements (management of waste) (range 0.06–5.02 mg/m^3^) exceeded the OEL of 5 mg/m^3^ for RD.

The calculated 8-h-TWA for RCS was 0.08 mg/m^3^ for all the measurements. In total 45 % of the calculated 8-h-TWA for the individual measurements (<0.02 to 0.24 mg/m^3^) exceeded the OEL of 0.1 mg/m^3^ for RCS.

Figure 1b shows the dust concentrations for TD and RD for demolition workers divided in different work task. When comparing the four work tasks differences in dust concentrations were found for TD (F = 18.9, df = 19, *p* < 0.001), but not for RD (F = 3.16, df = 10, *p* = 0.10). The difference between the work tasks for TD disappeared when the job tasks: ‘manual demolition’ and waste management were excluded from the analysis (F = 4.03, df = 10, *p* = 0.09).

TD was moderate correlated with RD (Spearman’s correlation *r*_s_ = 0.69), Fig. 2.

### Demolition workers compared to carpenters

The mean difference between TD for demolition workers and carpenters was 11.4 (95 % CI 3.46–37.1) mg/m^3^ (t = 4.26, df =56, *p* < 0.001). The mean difference between RD for demolition workers and carpenters was 3.90 (95 % CI 1.13–13.5) mg/m^3^ (t = 2.40, df =34, *p* = 0.03).

In linear regression models, TD was exp(2.43) = 11.4 (95 % CI 4.58–28.2) in demolition workers compared to carpenters unadjusted and exp(2.20) = 9.03 (95 % CI 4.00–20.3) adjusted for season. TD was exp(1.71) = 5.53 (95 % CI 2.39–12.7) in winter/spring compared to summer/autumn. Written in equation: logTD = ‐ 4.12 + 2.20 * occupation + 1.71 * season.

RD was exp(1.36) = 3.90 (95 % CI 1.65–9.23) for demolition workers compared to carpenters unadjusted and exp(1.24) = 3.46 (95 % CI 1.52–7.83) adjusted for season. RD was exp(1.08) = 2.94 (95 % CI 1.13–2.03) in winter/spring compared to summer/autumn.

Written in equation: logRD = ‐ 3.79 + 1.24 * occupation + 1.08 * season.

## Discussion

The results showed a more than ten-fold higher exposure to TD and four to five-fold higher exposures to RD for demolition workers compared to carpenters. The dust level depended on the work tasks and was highest for demolition workers during manual demolition and waste management and for carpenters during installing gypsum.

The calculated 8-h TWA of TD and RD were low for carpenters and below the OEL regardless of the work task (OEL are 10 mg/m^3^ for TD and 5 mg/m^3^ for RD in Denmark). The measurements showed very high concentrations especially of TD for demolition workers where the calculated 8-h TWA exceeded the OEL for 40 % of the measurements.

Wiebert et al. [11] assessed the exposure to dust for construction workers in Nordic countries by using job-exposure matrices and used levels of RCS of 0.01 mg/m^3^, inhalable dust of 1 mg/m^3^ and inhalable wood dust of 0.2 mg/m^3^ for all construction workers. In the present study we found much higher dust concentrations, but only for demolition workers, and large variations in dust exposure for the two occupations depending on work tasks.

Bagschik et al. [5] collected 100,000 measurements recorded by the German Institution for Statutory Accident Insurance and Prevention. The measurements were carried out from 1972 onwards and were divided into occupations of construction and different work tasks. The average exposure of RD was 0.96 mg/m^3^ (0.19–2.13 mg/m^3^) for installing gypsum. The dust concentrations varied according to the type of material, the strength of the material and the local conditions. They showed demolition work to be among the most dust-intensive of all work tasks in the construction industry. The average concentration of RD during mechanized demolition was 1.15 mg/m^3^ (0.25–2.67 mg/m^3^) and of RCS 0.12 mg/m^3^ (0.01–0.23 mg/m^3^). For manual demolition the concentration of RCS was 0.26 mg/m^3^. The highest concentrations occurred during demolition of concrete and reinforced concrete components, and when using high-speed handheld tools. Chrisholm [12] showed concentrations of RD due to demolition work between 1.3 mg/m^3^ when using a jack hammer and 4.7 mg/m^3^ when using a grinder. Karlsson and Christensson [7] reported concentration of RD of 7 mg/m^3^ for demolition work in 1975 to 1988 and 3 mg/m^3^ in 2005. Lumens and Spee [13] found concentrations of RD 10.8 to 14.4 mg/m^3^ when working with dry saw cutting. The concentration was reduced to 1.1 mg/m^3^ during wet cutting. The previous studies thus supported our findings of significant differences in the measured concentrations of RD depending on the profession, the work task and preventive measures.

The strength of this study was that the measurements were made after identification of the most relevant work tasks, included measurements for each work task and in different workers. Furthermore the measurements were conducted over an entire working day (excluding pauses) or as long as the task was done. The measurements also have been carried out on more workers performing the same work task at the same time and at the same workplace. Workplaces were not selected by level of dust (worst cases) but chosen if the work task was carried out.

The weakness of the study was no outdoor measurements which may result in an overestimation of the estimated dust exposure since the exposure outdoors are generally lower. Another weakness is a limited number of measurements especially for demolition workers.

Seasonal variations may cause a bias in the dust measurements if the performed work tasks and the ventilation through windows and doors changes during the seasons. In the analysis we therefore adjusted for eventually seasonal variations divided in winter/spring and summer/autumn. After adjustment for seasonal variations the results were only changed to a minor degree. Unfortunately, we had too few measurements to carry out statistical analysis in relation to each of the seasons which therefore may be a possible bias in this study.

The measurements showed only the current and not the previous level of dust exposure, where the level may have been higher, but most likely there have also previously been differences in the dust exposure between carpenters and demolition workers.

The literature has shown that prolonged exposure to high concentrations of RCS can cause silicosis, and studies have shown that COPD is associated with exposure to RCS [14, 15]. The risk of developing COPD depends, in general, on the level of dust exposure, on the duration of dust exposure per day and the number of years of exposure, in total. We do not know the level of exposure that affects the respiratory system or if there is a ‘no effect level’. Studies suggest that exposure beyond OEL may also result in an increased risk of COPD [14, 15]. In this study 45 % of the 8-h-TWA of RCS for demolition workers exceeded OEL (up to 2.4 times the Danish OEL of 0.1 mg/m^3^). The concentrations of RCS exceeded the OEL even though the RD level was below the OEL for RD. This emphasizes the importance of not only measuring the TD and RD but also supplementing these measurements with RCS if there is dust from concrete, mortar, gypsum or granite. It also supports the importance of using preventive measures such as water, local exhaust ventilation or respiratory protection. The measurements in this study support the practical experience and earlier measurements showing that different prevention strategies reduce the exposure and thus the risk of developing respiratory diseases [6].

Different sized particles deposit in different areas of the lung, nose and throat and may result in irritative symptoms including cough and COPD. The measurements of TD and RD concentrations are not directly comparable and there is no conversion factor to calculate the respirable fraction of dust by using measured TD concentrations. Studies report four times higher ‘total’ dust concentration compared to the respirable dust concentration among high-exposed cement workers [16, 17]. In a study from the cement industry RD, TD, and inhalable fractions were compared to thoracic fractions. The median ratios between the parameters’ were 0.51, 2.4 and 5.9, respectively. They found therefore that the cement industry would be able to predict the health related dust level by future measurements of just one of the fractions [18]. In this study the dust measurements for carpenters and demolition workers, showed a correlation between TD and RD but with large differences in the measured concentrations of TD compared to RD and RCS. Even at high concentrations of TD the concentration of RD was low, and despite relatively low concentrations of RD concentration of RCS was high and exceeded the OEL. In the construction industry it is therefore not enough in future measurements to measure one of the fractions to predict the health related dust level.

In the current study carpenters did not use respiratory protection and the demolition workers used respiratory protection in less than 1/3 of the dusty work processes and they may thus have been considerably exposed to dust at some of the work tasks. The current measurements represent the workers’ exposure to dust without using personal respiratory protection. Use of different types of personal equipment would reduce their exposure depending on the personal respiratory protection used (protection factor 5 to 50) [19].

Our study supports the importance to include different measurements of dust (e.g. TD, RD and RCS), for specific occupations and for work tasks where a dust exposure are expected, to use personal respiratory protection and other prevention measures.

## Conclusions

This measurement study showed differences in dust exposure between two professions within the construction industry. As expected, demolition workers were exposed to a much higher extent than carpenters. There was difference in dust exposure depending on profession, but also depending on work task for both occupations.
